# Degranulation Response in Cytotoxic Rat Lymphocytes Measured with a Novel CD107a Antibody

**DOI:** 10.3389/fimmu.2016.00572

**Published:** 2016-12-07

**Authors:** Amanda Sudworth, Ke-Zheng Dai, John T. Vaage, Lise Kveberg

**Affiliations:** ^1^Department of Molecular Medicine, Institute of Basic Medical Sciences, University of Oslo, Oslo, Norway; ^2^Department of Immunology, University of Oslo, Oslo University Hospital, Rikshospitalet, Oslo, Norway

**Keywords:** rat, lymphocyte, degranulation, functional assay, CD107a, cytotoxicity

## Abstract

Measuring degranulation through CD107a expression has become an advantageous tool for testing the functional capacity of cytotoxic cells. Such functional studies have been hampered in the rat by the lack of a suitable anti-rat CD107a antibody. In this study, we report a novel hybridoma generated by immunizing Armenian inbred hamsters with transfected Chinese hamster ovary cells expressing CD107a. The SIM1 clone exhibited specific reactivity with CD107a and measured degranulation from natural killer (NK) cells stimulated with target cells or mAb crosslinking of their activating receptors. Degranulation in IL-2-activated NK cells could also be measured, when using low effector to target ratios. SIM1 also stained activated CD8, but not CD4 T cells. This report characterizes the degranulation response in cytotoxic rat cells with a new antibody against rat CD107a.

## Introduction

Cytotoxic cells are integral in the fight against viral-infected and cancer cells. These cells kill through three different pathways, the release of lytic granules, Fas receptor-ligand pathway, or the TRAIL–TRAIL receptor pathway. For the purpose of this paper, we will focus on degranulation. When a cytotoxic cell encounters a target cell, it adheres tightly to it forming the immune synapse. The lytic granules are then polarized toward the immunologic synapse and released from the cytotoxic cell directed toward the target (1). These granules contain various cytotoxic mediators such as perforin and granzymes. Released perforin can form transient pores within the target cell membrane allowing granzymes to enter the target cell. Entry is probably also mediated by perforin oligomers forming incomplete pores (arcs) (2), and it has also been suggested that granzymes and perforin can be taken up into the target cells through the endosomal pathway (3, 4). The data behind these proposed mechanisms have recently been discussed in detail (5). When granzymes enter target cells the caspase pathway is activated resulting in apoptosis (6, 7). The membrane of these cytotoxic granules contains lysosomal-associated membrane glycoprotein-1 (LAMP1), also known as CD107a (8). The luminal part of rat CD107a is transiently exposed to the extracellular side of the cell membrane after degranulation before it is again internalized. Therefore, CD107a staining indicates that a degranulation event has occurred (9). While the role for CD107a is still being determined, siRNA studies against CD107a has shown it is essential for perforin trafficking within the vesicles, as delivery of perforin to lytic granules is inhibited in the absence of CD107a (10). CD107a has also been shown to have a role in protecting cytotoxic cells from death by binding to perforin and preventing insertion into the cell membrane (11).

There are two main types of cytotoxic lymphocytes, natural killer cells (NK) and CD8 T cells. Both cell types kill through the release of lytic granules, the Fas receptor/Fas ligand pathway and the TRAIL/TRAIL receptor pathway, but the stimulus necessary to activate these two cell groups is different. T cells recognize antigen presented by MHC class I molecules through their activating T cell receptor (TCR), while NK cells recognize target cell antigens though various inhibitory and activating receptors. Inhibitory receptors are important for preventing NK cells from killing healthy cells while activating receptors induce NK cell cytotoxicity.

Before the identification of CD107a as a marker for degranulation in NK cells (12), the output for measuring the cytotoxic ability was the death of the target cells. Detection of CD107a by flow cytometry allowed researchers to identify the specific effector cells with cytotoxic ability. Since its discovery, a wide array of publications of data from mouse and human studies has been completed showing the advantage of using an anti-CD107a antibody. The rat is a valuable immunological animal model for studying diseases such as leukemia and graft vs. host disease (13), but functional readouts have been hampered by the lack of high quality specific anti-rat CD107a antibodies for flow cytometry. This paper describes the creation of an antibody toward rat CD107a and characterization of the degranulation response in rat NK and CD8^+^ T cells.

## Materials and Methods

### Animals

Armenian inbred hamsters (Cytogen Research and Development Inc., MA, USA) were housed in the Department of Comparative Medicine at Rikshospitalet, Oslo University Hospital, in agreement with institutional guidelines. The rat strains PVG-RT1U, PVG-RT7b, PVG-RT1N, and PVG-R23 were used interchangeably and maintained for more than 20 generations and housed in compliance with guidelines set by the Experimental Animal Board under the Ministry of Agriculture of Norway and by the European Convention for the Protection of Vertebrate Animals used for Experimental and Other Scientific Purpose. Rats were used by the age of 8–12 weeks. The laboratory animal facilities are subjected to a routine health-monitoring program and tested for infectious organisms according to a modification of Federation of European Laboratory Animal Science Association recommendations. Animal experiments were regulated through laboratory regulations, which are put forth by the Animal Welfare act in which Norway follows the EU laboratory direction and the European laboratory convention.

### Abs and Reagents

Antibodies against NKp46 [Wen23-Pacific Blue (PB) or unconjugated], NKR-P1A 3.2.3-PB, or unconjugated (binding only NKR-P1A, but not NKR-P1B, in the PVG strain), CD8 (OX8-biotin), CD2 (OX34, unconjugated), TCR (R73-Alexa647), mouse IgD b allotype (TIB96-unconjugated, used as mouse IgG isotype), and CD45.2 (His41-FITC, used as mouse IgG isotype) were made in our laboratory and conjugated according to standard protocols. Antibodies against CD3 (IF4, unconjugated) and its isotype mouse IgM (MM-30, unconjugated) and Armenian hamster IgG isotype control (HTK88-FITC) were purchased from Biolegend (San Diego, CA, USA). Commercial anti-CD107a antibodies were purchased from AbCam (Cambridge, UK) (H4A3-FITC), eBioscience (San Diego, CA, USA) (ID48-PerCP-eFluor), and Lifespan (Seattle, WA, USA) (LS-C8580-unconjugated). LS-C8580 was conjugated with Alexa488 NHS ester (Sigma-Aldrich, St. Louis, MO, USA) for use in functional assays. Anti-FLAG antibody (M2, unconjugated) and anti-mouse IgG (goat polyclonal-FITC) were purchased from Sigma-Aldrich (St. Louis, MO, USA). IL-2 was obtained from a dialyzed cell culture supernatant of a Chinese hamster ovary (CHO) cell line stably transfected with a rat IL-2 expression construct. Peridinin Chlorophyll-a Protein (PerCP) conjugated streptavidin, FITC hamster IgG polyclonal isotype control, FITC mouse IgG1 isotype control (MOPC-FITC), and GolgiStop were purchased from BD biosciences (Franklin Lakes, NJ, USA). Goat anti-Armenian hamster (polyclonal-FITC) was purchased from Jackson Immunoresearch (West Grove, PA, USA). Supernatants from hybridomas OX41, CD172a (SIRPα), R73 (anti-rat TCRαβ), OX19 (anti-CD5), OX12 (anti-Igκ chain), and OX33 (anti-CD45RA) were made in-house. Control hamster serum was derived from un-immunized Armenian hamsters.

### Cloning of rat CD107a

Rat CD107a was PCR amplified from cDNA generated from PVG rat splenocytes and subcloned into a pMX expression vector containing a puromycin resistance gene (a generous gift from Dr. Michael R. Daws) (14). CD107 primers were designed based on a rat CD107a (LAMP1) cDNA sequence (GenBank: NM_012857). Forward primer: 5′-GCCCCAGCACTGTTCGAGGT-3′; Reverse primer: 5′-GTTGTTACCGTCCTGTACACACT-3′. An anionic trypsin-1 leader peptide (MSALLILALVGAAVA) was used in the expression vector, and the original CD107 transmembrane region was replaced by a CD8 transmembrane fragment to facilitate high surface expression. A FLAG-tag (DYKDDDDK) was ligated to the N-terminal of the CD107 protein to make the surface expression detectable. All inserted sequences and open reading frame in the final chimeric plasmid construct were examined and confirmed by Sanger DNA sequencing.

### Transfecting BWZ.36 Cells with pMX-CD107a Plasmid

293T cells were seeded into a 6-well cell culture plate with 4 ml RPMI 1640 medium, transfected with 7.5 μl FuGENE6 reagent (Roche, manufacturer’s protocol) and incubated at 37°C with 5% CO_2_. The FuGENE6 reagent was pre-diluted with *Opti-MEM* (Gibco) and mixed with 1.5 μg pMX-CD107a plasmid and 1 μg pCL-ECO rodent specific retroviral packaging plasmid (Addgene #12371). Twenty-four hours after transfection, the supernatant was descanted and replaced with RPMI1640 medium with 2% FBS and transferred to 32°C for retroviral production. Forty-eight hours later, the supernatant was harvested and filtered with a 0.2-μm Filtropur S filter (Sarstedt) and saved at −80°C for retroviral transduction.

The 10^5^ BWZ.36 cells [Dr. Nilabh Shastri, U.C. Berkeley, CA, USA (15)] were seeded per well into a 24-well cell culture plate (Corning) and cultured with fresh RPMI1640 medium with 10% FBS, overnight. The supernatant was removed and RPMI1640 containing 10% FBS, 16 μg polybrene (to a final concentration 8 μg/ml), and 1.5 ml retroviral supernatant was added and centrifuged at 32°C at 2400 rpm for 2 h. The cells were then incubated at 32°C for 1 h after which the supernatant was removed and replaced with fresh RPMI1640 medium with 10% FBS and cultured for 3 days, at 37°C with 5% CO_2_ (on day 2, complete medium with 1 ng/ml puromycin could be used). Expanded BWZ.CD107a-FLAG transfectants were examined for surface expression of the FLAG-tag and then were used for antibody immunization or antibody screening.

### Generating CD107a Expressing Transfectants

The chimeric rat CD107a plasmid was transfected into CHO cells. Briefly, CHO cells were cultured in complete RPMI1640 medium (cRPMI; RPMI1640 medium with 10% FBS, 1mM Na Pyruvate, 50μM 2-mercaptoethanol) until they reached 30% confluency. The 2.5 μg plasmid was mixed with 7.5 μl of Fugene 6 reagent (Roche) [pre-diluted with *Opti-MEM*^®^ (Gibco)] and added onto cells dropwise. Two days following, the CHO cells were detached with RPMI1640 medium supplemented with 2mM EDTA and were seeded out in 96-well flat bottom cell culture plates (25 cells/well) with selection medium (cRPMI supplemented with 20 μg/ml puromycin) for cloning of stable transfectants. After 10–12 days culture, grown-up clones were screened with anti-FLAG antibody (M2). One of the clones expressing highest surface level of FLAG and CD107a was used for immunizations.

### Immunization and Cell Fusion

The 3- to 10-week-old male Armenian inbred hamsters were immunized by intraperitoneal injections (i.p.) with 2 × 10^6^ CHO.CD107a-FLAG cells once a week for 3 weeks. A boost with 4 × 10^6^ stably transfected BWZ.CD107a-FLAG cells was given i.p. 32 days after first immunization and the sera were analyzed for surface staining of both CHO.CD107a-FLAG and BWZ.CD107a-FLAG transfectants. Two of the three animals were positive, and 3 days after boosting, spleen cells were harvested and pooled from the two animals, one of which had splenomegaly. About 2.0 × 10^8^ spleen cells and 1.4 × 10^8^ NS0 myeloma cells were washed three times in DMEM with additives (1mM sodium pyruvate, 0.05mM 2-mercaptoethanol and antibiotic/antimycotic) and centrifuged at 90 *g* for 10 min. Keeping everything at 37°C from this point forward, the pellet was dissolved by tapping the tube and prewarmed polyethylene glycol 1500 (Roche) was added drop by drop while gently stirring for 1 min. Three milliliters of DMEM with additives were gradually added for the next 3 min finishing with an extra 7 ml before centrifuged at 500 *g*. The pellet was washed by careful flushing (no resuspension) with DMEM with additives and 20% FBS (DMEM-20) and cultured overnight. Cells were harvested the following day and 100 ml DMEM-20 with 2× hypoxanthine–aminopterin–thymidine (SIGMA) and 10% hybridoma cloning supplement (HCS) (PAA) was added. The cell suspension was transferred to 96-well plates. After 12 days, supernatants from growing clones were tested by flow cytometry for staining of surface antigens present on BWZ.CD107a-FLAG and CHO.CD107a-FLAG, but not on BWZ.36 cells transfected with irrelevant FLAG-tagged antigens as negative controls. The 9 of 12 positive clones producing specific antibodies were further subcloned using DMEM-20 supplemented with hypoxanthine-thymidine and HCS. Only one clone (SIM1) still produced antibodies after subcloning and this was further subcloned one more time. CD107a specific hamster IgG antibodies were detected by FITC anti-Armenian hamster IgG (Jackson Immunoresearch). Anti-CD107a from the SIM1 hybridoma was purified by HiTrap Protein G HP (GE Healthcare, Life Sciences) and FITC-conjugated according to standard procedures or Alexa488-conjugated according to manufacturer’s protocol.

### Generation of Effector Cells

Single cell suspensions were prepared from the spleen. Lymphocytes were isolated using a Lymphoprep gradient and then run over a nylon wool column to remove B cells and macrophages. For generation of IL-2-activated NK cells (LAK), the remaining cells were cultured overnight in IL-2. The following day, non-adherent cells were removed by washing with PBS and the adherent NK cells were maintained in RPMI supplemented with IL-2, 10% heat inactivated FBS, 1% Streptomycin/Penicillin, 1mM sodium pyruvate, and 50 μM 2-mercaptoethanol. Cells were cultivated for 8–10 days. For generation of enriched NK cells or sorted T and NK cells, cells were enriched by negative selection after the nylon wool step. For enrichment of NK cells, pan-mouse IgG Dynabeads (Invitrogen Dynal) were coated with antibodies against macrophages (OX41), T cells (R73 and OX19), and B cells (OX12 and OX33). For enrichment of T cells, pan-mouse IgG Dynabeads were stained with antibodies against macrophages (OX41) and B cells (OX12 and OX33). Cells were enriched by two steps of negative selection with stained beads for 30 min at 4°C. Enriched NK cells had a purity of approximately 50–60% and were used for degranulation studies. Enriched T cells (79–85% purity) were further sorted for TCR^+^ cells using a FACS Aria (BD Biosciences).

### Degranulation Assays

To measure the degranulation response by staining with anti-rat CD107a (SIM1) during target cell stimulation, NK effectors and YAC targets cells were incubated, at a 1 to 1 ratio of 3 × 10^5^ cell/well (unless stated otherwise) in media containing the SIM1 mAb, the other commercially available anti-CD107a antibodies, or isotype control. To measure the degranulation response to antibody stimulation from NK cells, we used flat bottom 96-well plates coated with varying concentrations of anti-NKp46 (Wen23), anti-NKR-P1A (3.2.3), and a mouse IgG1 isotype control (TIB96). The following day, plates were washed with PBS and blocked with cRPMI before addition of 3 × 10^5^ enriched NK cells suspended in cRPMI containing SIM1 were added per well. To measure degranulation from T cells, they were sorted and cultured in cRPMI with IL-2 for 2–3 days. The 96-well flat bottom plates were coated over night with antibodies against CD3 (IF4), CD2 (OX34), or an IgM isotype control (MM-30). About 3 × 10^5^ T cells were added per well in cRPMI containing SIM1. All assays were incubated for 4 h. Golgistop was added for the last 3 h of incubation to prevent the degradation of internalized CD107a. Cells were then washed and stained with antibodies toward the appropriate surface receptors and analyzed using the FACS Canto or LSR II (BD Biosciences).

### Chromium Release Assay

YAC-1 target cells (5 × 10^6^ cells/ml cRPMI) with added 3.7 MBq^51^Cr were incubated at 37°C for 1 h, stirring every 15 min. Cells were then washed three times with PBS with 2% FBS and resuspended to 1 × 10^5^ cells/ml in cRPMI. LAK effector cells and target cells were plated at the specified ratios in a final volume of 200 μl. Cells were centrifuged and incubated for 4 h at 37°C. Specific release of ^51^Cr was measured using the Cobra auto-gamma (Packard).

## Results

**Figure 1:**
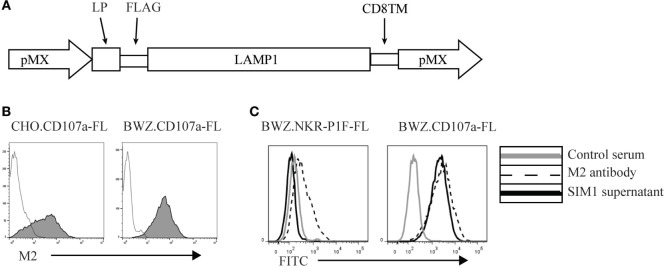
**Generation of an anti-rat CD107a monoclonal antibody (SIM1)**. **(A)** Schematic vector map of the pMX expression vector with leader peptide, FLAG-tag, CD107a insert, and the transmembrane domain of CD8. **(B)** Stably transfected Chinese hamster ovary and BWZ.36 cells were tested for cell surface expression of FLAG-tagged CD107a using an anti-FLAG antibody (M2) (shaded histograms). Negative control is secondary antibody alone (transparent histogram). **(C)** BWZ cells transfected with CD107a-FLAG or irrelevant antigen (NKR-P1F-FLAG) were stained with hamster control serum, SIM1 supernatant, or an anti-FLAG antibody (M2).

To create an antibody against CD107a, we made an expression vector containing the extracellular membrane region of rat CD107a, the transmembrane region of rat CD8, and a 3′-terminal FLAG-tag to detect surface expression (Figure 1A). The vector was transfected into CHO cells and the BWZ.36 T cell line and screened for CD107a expression using an anti-FLAG antibody (M2) (Figure 1B). CD107a stably transfected CHO cells were injected into Armenian inbred hamsters once a week for 3 weeks with a final boost of CD107a stably transfected BWZ cells 3 days before fusion with the NS0 myeloma cell line. Supernatants from the different clones were tested for specificity toward BWZ.CD107a-FLAG, but not irrelevant FLAG-tagged BWZ.36 transfectants. One clone, SIM1, was found to bind specifically to the CD107a expressing transfectant, but not to the FLAG transfected control (Figure 1C).

**Figure 2:**
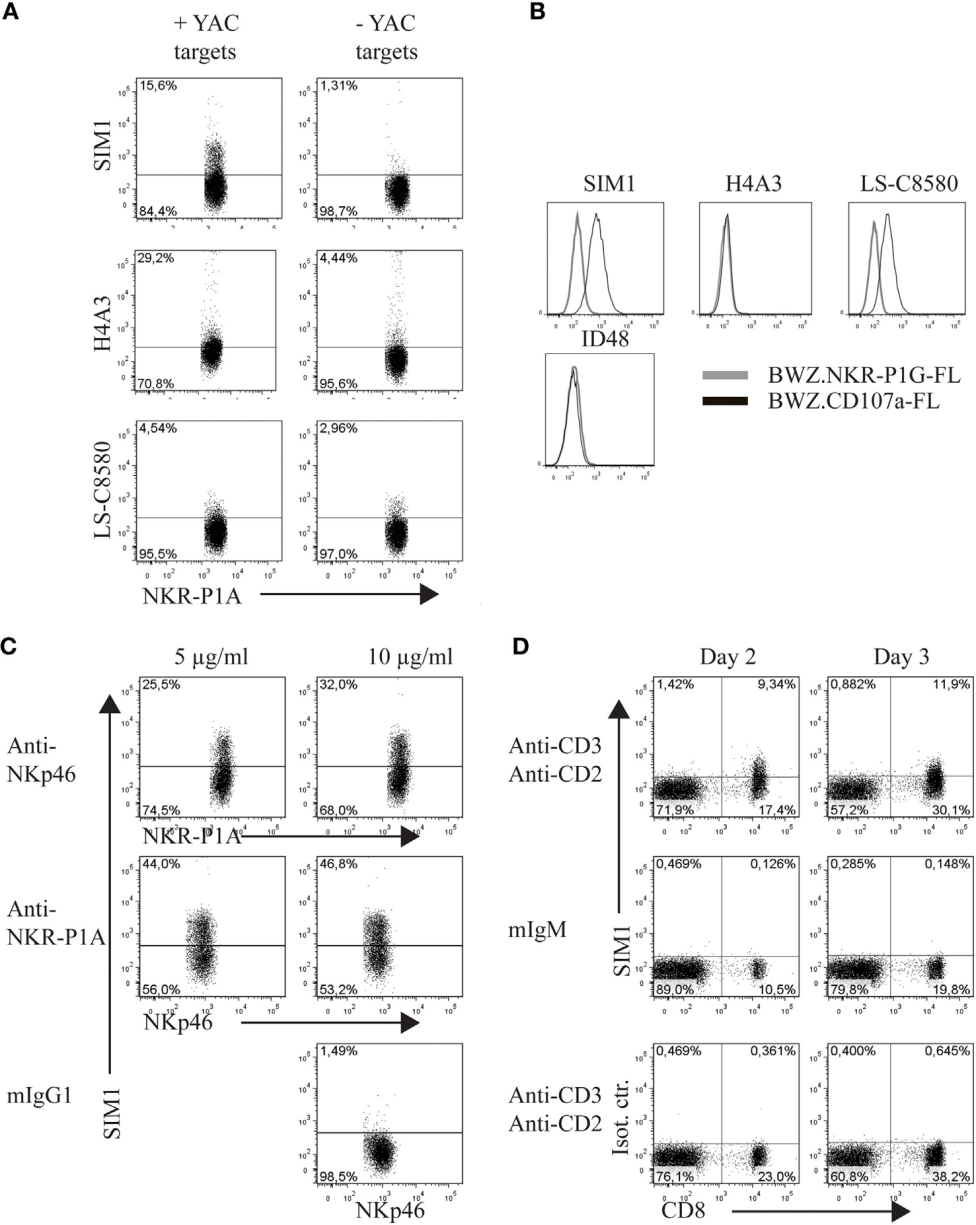
**SIM1 measures degranulation from natural killer (NK) and CD8 T cells**. **(A)** Percent degranulating cells were measured using anti-CD107a antibodies SIM1, H4A3, and LS-C8580, with or without YAC-1 target cells as a stimulus. One representative experiment of four is shown **(B)**. Staining of the BWZ.CD107a-FLAG cell line with SIM1, H4A3, LS-C8580, or ID48 antibody clones. BWZ.36 cells transfected with irrelevant FLAG-tagged antigen (BWZ.NKR-P1G-FLAG) was included as a negative control, **(C)** enriched NK cells were stimulated with plate bound anti-NKp46, anti-NKR-P1A, or mouse IgG1 as isotype control, and SIM1 was used to measure percentage degranulating cells. NKp46^+^ (Wen23) or NKR-P1A^bright^ (3.2.3) cells were gated on. **(D)** TCR^+^ cells were sorted (purity 99%) and cultured in IL-2 for 2–3 days. The cells were then stimulated with plate bound anti-CD3 and anti-CD2 or mouse IgM isotype control for anti-CD3. The lower panel shows stimulation with anti-CD3 and anti-CD2 and staining with a SIM1 isotype control (FITC hamster IgG polyclonal). T cells were sorted using R73-Alexa647 and 3.2.3-PB antibodies, gating on R73^+^/3.2.3^−^ cells. Data shown are from one representative experiment of three separate independent experiments.

The clone SIM1 was further tested in functional assays in parallel with two commercially available CD107a antibodies, which could potentially cross react with rat CD107a in flow cytometry. The antibody clones tested were H4A3 from AbCam (which cross reacts with human, rat, mouse, and primates in flow cytometry) and the antibody clone LS-C8580 from LifeSpan BioSciences (which cross reacts with CD107a from mouse, human and rats in immunohistochemistry and Western blots according to the manufacturer’s website). Our SIM1 antibody clone identified degranulating cells in the presence of target cells and showed little background staining in the absence of targets (upper panel Figure 2A). Clone H4A3 stained the highest percentage of NK cells after target cell stimulation (middle panel). However, there was also a high level of background staining in the absence of target cells, indicating unspecific binding. Very low levels of degranulation, if any, were measured by the LS-C8580 mAb clone (lower panel). The specificity of these antibodies was confirmed by positive staining of the BWZ.CD107a-FLAG transfectant, but not BWZ.NKR-P1F-FLAG control (Figure 2B). The antibody clone H4A3, which has been shown to bind to human CD107a (16), did not stain rat CD107a transfected BWZ cells, indicating there is no cross reaction with rat CD107a. The anti-mouse CD107a clone ID48 also failed to stain the rat CD107a transfectant. Unconjugated LS-C8580, on the other hand, showed specific staining of CD107a transfected cells (data not shown). However, the LS-C8580 clone is only available as unconjugated antibody which is not suited for degranulation studies. Repeated attempts in our laboratory to conjugate LS-C8580 to Alexa488 have unfortunately not provided any strong staining conjugates, though the small sample size and concentration of LS-C8580 may be the cause of the poor conjugation. Conjugated LS-C8580 shows reduced but specific staining of CD107a transfected cells (Figure 2B), but as shown in Figure 2A, we were not able to detect degranulating cells during functional assays with this conjugate. In conclusion, the SIM1 mAb specifically stains degranulating rat NK cells after stimulation with target cells.

Direct stimulation of activating NK receptors through plate bound antibodies is another potent method to induce degranulation. To test the SIM1 clone in this functional assay, we incubated enriched NK cells on plates coated with antibodies toward the activating receptors NKp46 and NKR-P1A (Figure 2C). As a negative control, plates were coated with mouse IgG1 isotype control. Staining with the SIM1 antibody identified 25.5 or 44% degranulating cells after NK cell stimulation in plates coated with 5 μg/ml anti-NKp46 or anti-NKR-P1A respectively. The percentage of degranulating cells increased even further when the plates were coated with a higher concentration of stimulating antibodies (10 μg/ml). No degranulation was induced in wells coated with the mouse IgG1 isotype control.

We clearly saw degranulation in NK cells stimulated with either target cells or with antibodies against activation receptors by using our SIM1 antibody. We also wanted to confirm this in cytotoxic T cells. T cells were sorted from spleen cells and cultured in IL-2 for 2–3 days. The T cells were then stimulated with plate bound anti-CD3 and anti-CD2 (Figure 2D). Degranulation was measured from cultured CD8^+^ T cells when stimulated with anti-CD3 and anti-CD2 on both days 2 and 3. No degranulation was measured from non-cytotoxic CD4 T cells, or after stimulation with isotype control antibody, further demonstrating the specificity of our SIM1 antibody.

**Figure 3:**
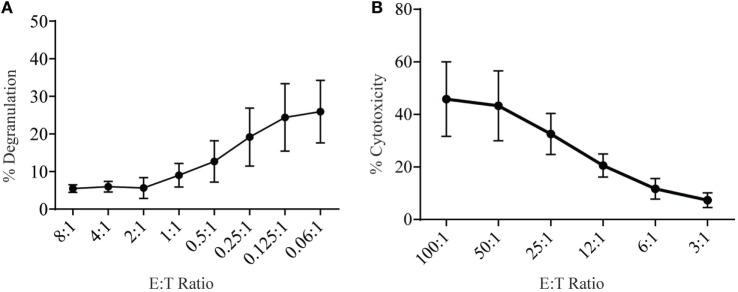
**Low effector:target ratios are important to ensure adequate sensitivity in degranulation assays with rat NK cells**. **(A)** Degranulation of LAK cells cultured 8–10 days in IL-2 against YAC-1 targets at different effector:target ratios. **(B)** Cytotoxicity of IL-2 cultured LAK against YAC-1 target cells was confirmed with the chromium release assay completed on days 8–10. All graphs are cumulative of three or more separate experiments with bars showing SD.

Lymphokine-activated killer (LAK) cells are created by culturing NK cells in IL-2 for 7–10 days and are commonly used as very potent effector cells in chromium release assays to study cytotoxicity and, therefore, should be able to generate high levels of degranulation. To measure degranulation from LAK cells, we initially used a 1:1 ratio of effector to targets but found unexpectedly low levels of degranulation (data not shown). However, doing a titration to find optimal effector to target ratios we saw increasing levels of degranulation could be achieved when effector to target ratios were below 1 (Figure 3A). The optimal ratio for degranulation assays is therefore in contrast to cytotoxicity assays where the level of cytotoxicity increases with higher effector: target ratios (Figure 3B).

## Discussion

In the initial testing of SIM1 and the different commercial CD107a antibodies in degranulation assays in the rat, we chose a small range of effector to target ratios with a skewing toward more effector cells than targets based on current literature (12, 16, 17). Effector to target cell ratios can vary from paper to paper, but previous studies which titrated effector to target ratios have shown that optimal degranulation from mouse NK cells peaked at 4.5:1 ratio with YAC-1 target cells (18) and human NK cells killed K562 cells best at a ratio of 5:1 or 10:1 (12). However, when using a larger range of effector to target ratios, we show that degranulation using rat LAK cells was more effectively induced when using four or more targets per effector cell. This difference in optimal effector:target ratio between the rat and other species could be due to the type of target cells used, as the human and mouse effector to target ratio titrations in the abovementioned studies used target cells that originated from the same species. The effector cells might be more sensitive to target cells from the same species and therefore need fewer target cells to become activated. The YAC-1 cell line used in our study is a mouse T cell lymphoma, which have been the standard for many years to stimulate cytotoxicity from rat NK cells. We cannot exclude that using a rat target cell would result in stronger stimulation of the NK cells and subsequently fewer target cells might be needed to induce degranulation.

It is also clear from this study that the effector to target ratios used for cytotoxicity assays do not correlate well with the ratios needed to measure optimal degranulation response in the effector cells. This is not surprising as the read out for these two assays are different, with cytotoxicity measuring the target cell death, and degranulation measuring the NK response to targets.

In conclusion, we have raised an antibody against rat CD107a (SIM1) and characterized its ability to detect degranulation in cytotoxic lymphocytes in the rat. We have shown that SIM1 is specific to degranulating cells, and through optimization of the degranulation protocol, we have determined that using effector:target cell ratios lower than 1:4 ensure optimal stimulation of LAK cells. The development of anti-rat CD107a (SIM1) allows better analysis and characterization of the cytotoxic responses of rat lymphocytes and will be a valuable tool in the many disease models of the rat.

## Ethics Statement

The study is approved by the National Animal Research Authority (FDU), Norway (permit number: ID-1698).

## Author Contributions

AS has been responsible for the expression analysis, functional assays, and writing of the manuscript. K-ZD has performed the cDNA cloning and generation of transfectants. JTV has been involved in the project planning and supervision, and in the writing of the manuscript. LK has been responsible for immunizations, the hybridoma technology, writing of the manuscript, and project supervision.

## Conflict of Interest Statement

The authors declare that the research was conducted in the absence of any commercial or financial relationships that could be construed as a potential conflict of interest.

## References

[1] Orange JS. Formation and function of the lytic NK-cell immunological synapse. Nat Rev Immunol (2008) 8(9):713–25. 10.1038/nri2381 19172692 PMC2772177

[2] Metkar SS Marchioretto M Antonini V Lunelli L Wang B Gilbert RJ Perforin oligomers form arcs in cellular membranes: a locus for intracellular delivery of granzymes. Cell Death Differ (2015) 22(1):74–85. 10.1038/cdd.2014.110 25146929 PMC4262768

[3] Masson D Tschopp J. Isolation of a lytic, pore-forming protein (perforin) from cytolytic T-lymphocytes. J Biol Chem (1985) 260(16):9069–72. 3874868

[4] Thiery J Keefe D Boulant S Boucrot E Walch M Martinvalet D Perforin pores in the endosomal membrane trigger the release of endocytosed granzyme B into the cytosol of target cells. Nat Immunol (2011) 12(8):770–7. 10.1038/ni.2050 21685908 PMC3140544

[5] Voskoboinik I Whisstock JC Trapani JA. Perforin and granzymes: function, dysfunction and human pathology. Nat Rev Immunol (2015) 15(6):388–400. 10.1038/nri3839 25998963

[6] Chowdhury D Lieberman J. Death by a thousand cuts: granzyme pathways of programmed cell death. Annu Rev Immunol (2008) 26:389–420. 10.1146/annurev.immunol.26.021607.090404 18304003 PMC2790083

[7] Lopez JA Brennan AJ Whisstock JC Voskoboinik I Trapani JA. Protecting a serial killer: pathways for perforin trafficking and self-defence ensure sequential target cell death. Trends Immunol (2012) 33(8):406–12. 10.1016/j.it.2012.04.001 22608996

[8] Peters PJ Borst J Oorschot V Fukuda M Krahenbuhl O Tschopp J Cytotoxic T lymphocyte granules are secretory lysosomes, containing both perforin and granzymes. J Exp Med (1991) 173(5):1099–109. 10.1084/jem.173.5.1099 2022921 PMC2118839

[9] Betts MR Brenchley JM Price DA De Rosa SC Douek DC Roederer M Sensitive and viable identification of antigen-specific CD8+ T cells by a flow cytometric assay for degranulation. J Immunol Methods (2003) 281(1–2):65–78. 10.1016/S0022-1759(03)00265-5 14580882

[10] Krzewski K Gil-Krzewska A Nguyen V Peruzzi G Coligan JE. LAMP1/CD107a is required for efficient perforin delivery to lytic granules and NK-cell cytotoxicity. Blood (2013) 121(23):4672–83. 10.1182/blood-2012-08-453738 23632890 PMC3674668

[11] Cohnen A Chiang SC Stojanovic A Schmidt H Claus M Saftig P Surface CD107a/LAMP-1 protects natural killer cells from degranulation-associated damage. Blood (2013) 122(8):1411–8. 10.1182/blood-2012-07-441832 23847195

[12] Alter G Malenfant JM Altfeld M. CD107a as a functional marker for the identification of natural killer cell activity. J Immunol Methods (2004) 294(1–2):15–22. 10.1016/j.jim.2004.08.008 15604012

[13] Nestvold JM Omdal BK Dai KZ Martens A Benestad HB Vaage JT A second prophylactic MHC-mismatched bone marrow transplantation protects against rat acute myeloid leukemia (BNML) without lethal graft-versus-host disease. Transplantation (2008) 85(1):102–11. 10.1097/01.tp.0000296856.53493.1f 18192919

[14] Onishi M Kinoshita S Morikawa Y Shibuya A Phillips J Lanier LL Applications of retrovirus-mediated expression cloning. Exp Hematol (1996) 24(2):324–9. 8641361

[15] Sanderson S Shastri N. LacZ inducible, antigen/MHC-specific T cell hybrids. Int Immunol (1994) 6(3):369–76. 10.1093/intimm/6.3.369 8186188

[16] Murugin VV Zuikova IN Murugina NE Shulzhenko AE Pinegin BV Pashenkov MV. Reduced degranulation of NK cells in patients with frequently recurring herpes. Clin Vaccine Immunol (2011) 18(9):1410–5. 10.1128/CVI.05084-11 21734066 PMC3165235

[17] Bryceson YT Fauriat C Nunes JM Wood SM Björkström NK Long EO Functional analysis of human NK cells by flow cytometry. Methods Mol Biol (2010) 612:335–52. 10.1007/978-1-60761-362-6_23 20033652 PMC4969010

[18] Vahlne G Becker S Brodin P Johansson MH. IFN-gamma production and degranulation are differentially regulated in response to stimulation in murine natural killer cells. Scand J Immunol (2008) 67(1):1–11. 10.1111/j.1365-3083.2007.02026.x 18028287

